# Supplementary material for: Response to Nodal morphogen gradient is determined by the kinetics of target gene induction
Source: eLife. 2015 Apr 14;4:e05042. doi: 10.7554/eLife.05042 (PMC4395910; doi:10.7554/eLife.05042)
Supplement: Supplementary file 1. — Scripts used for tracking cells and measuring the nucleo-cytoplasmic ratio. DOI: http://dx.doi.org/10.7554/eLife.05042.030 [file elife05042s009.rtf]

Below are the MATLAB codes used for tracking and measuring the nucleocytoplasmic ratio of GFP-Smad2/Histone-RFP embryonic cells in zebrafish blastulae from time-lapse confocal images.The first code segments the images and extract positions and NC ratio. The second code cleans and formats the data for tracking. The third code tracks cells using a nearest neighbors strategy, the fourth code removes duplicated cells and rearranges the data, the fifth code defines the margin position using a GUI and the sixth code measures the distance of each cell at each time point from the margin.The final dataset is an n-by-m array of structures. Each structure contains data for one tracked cell. Each column corresponds to one frame of the movie. Tracked cells appearing in the ith frame is added up in the ith column.Each field 'cell' of the structure is a p-by-q matrix.1st column: frame number2nd column: x coordinate3rd column: y coordinate4th column z coordinate (slice number)5th column: NC ratio6th column: Distance traveled (microns) between each frame7th column: Distance from the margin(Other columns are not relevant)datasets:'trackedcells1' corresponds to data related to figure 2D and Fig2- Figure supplement 1'trackedcells2' corresponds to data related to figure 2E-G.These are 2 different embryos.1- Image segmentation and statistics (NC ratio …)% get stats from stacks, get NC ratio from slices where nucleus area is the% biggest, try to increase cell resolution for segmentation before tracking %% create a structure that contains all GFPSmad2 (GS) and HistoneRFP(HR)...% ...stacks for each framefunction stacks=getstatsstacksv4 %diary on %cd('/Users/Julien/Desktop/analyzed confocal data/MatLab figures/GSHRCelltracking');fn=numel(dir('GS0*.tif'));sln=input('How many slices?');fr=fn/sln;frd=input(['How many frames out of ' int2str(fr) ' total?']);tic stacks=struct([]);sgs=struct([]);shr=struct([]);for i=1:frd    for j=1:sln        dig=(i-1)*sln+(j-1);        if dig<10            tag=['000' int2str(dig)];        elseif dig>=10 && dig<100            tag=['00' int2str(dig)];        else            tag=['0' int2str(dig)];        end        GS=imread(['GS' tag '.tif']);        [ypix xpix]=size(GS);        sgs(j,1).gs=GS;        shr(j,1).hr=imread(['HR' tag '.tif']);    end    stacks(i,1).GS=cat(4,sgs.gs);    stacks(i,1).HR=cat(4,shr.hr);end %% threshold each HisRFP images, clean up out of focus ones, identify% nuclei in GFPSmad2 images and get stats for k=1:length(stacks)    H=im2bw(stacks(k,1).HR,graythresh(stacks(k,1).HR));    for ss=1:sln        H(:,:,ss)=bwareaopen(H(:,:,ss),20);    end    Hint=uint16(H);    Hintt=uint8(H);    HRS=Hintt.*stacks(k,1).HR;    GSnuc=Hint.*stacks(k,1).GS;    Hinv= Hint==0;    Hinv=uint16(Hinv);    GScyto=Hinv.*stacks(k,1).GS;    GScytot=im2bw(GScyto,graythresh(GScyto));    GScytot=uint16(GScytot);    GScyto=GScytot.*GScyto;    for ln=1:sln        Htemp=HRS(:,:,ln);        [BH,LH]=bwboundaries(Htemp,'noholes');        GSnuctemp=GSnuc(:,:,1,ln);        GScytotemp=GScyto(:,:,1,ln);        [B,L,N,A]=bwboundaries(GSnuctemp,'noholes');        stacks(k,1).Framestats(ln,1).stats=regionprops(L,'area','centroid','pixellist','pixelidxlist','convexarea');        stacks(k,1).Framestats(ln,1).Hstats=regionprops(LH,'pixelidxlist');        for nn=1:length(stacks(k,1).Framestats(ln,1).Hstats)            stacks(k,1).Framestats(ln,1).Hstats(nn).PixelValues=Htemp(stacks(k,1).Framestats(ln,1).Hstats(nn).PixelIdxList);            stacks(k,1).Framestats(ln,1).Hstats(nn).MeanIntensity=mean(stacks(k,1).Framestats(ln,1).Hstats(nn).PixelValues);        end         meancat=cat(1,stacks(k,1).Framestats(ln,1).Hstats.MeanIntensity);        meanmeancat=mean(meancat);         % solve discrepency between GS stats and HR stats        lgss=length(stacks(k,1).Framestats(ln,1).stats);        lhrs=length(stacks(k,1).Framestats(ln,1).Hstats);        if lgss>lhrs            stacks(k,1).Framestats(ln,1).stats((lhrs+1):end)=[];        elseif lhrs>lgss            stacks(k,1).Framestats(ln,1).Hstats((lgss+1):end)=[];        end          for mm=1:length(stacks(k,1).Framestats(ln,1).stats)            stacks(k,1).Framestats(ln,1).stats(mm).rap=(stacks(k,1).Framestats(ln,1).stats(mm).Area)/(stacks(k,1).Framestats(ln,1).stats(mm).ConvexArea);            stacks(k,1).Framestats(ln,1).stats(mm).PixelValues=GSnuctemp(stacks(k,1).Framestats(ln,1).stats(mm).PixelIdxList);            stacks(k,1).Framestats(ln,1).stats(mm).MeanIntensity=mean(stacks(k,1).Framestats(ln,1).stats(mm).PixelValues);            stacks(k,1).Framestats(ln,1).stats(mm).Roundoid=round(stacks(k,1).Framestats(ln,1).stats(mm).Centroid);            stacks(k,1).Framestats(ln,1).stats(mm).Slice=ln;            stacks(k,1).Framestats(ln,1).stats(mm).HRmI=stacks(k,1).Framestats(ln,1).Hstats(mm);        end        %ridrap=find([stacks(k,1).Framestats(ln,1).stats.rap]<0.9);          ridareamin=find([stacks(k,1).Framestats(ln,1).stats.Area]<50);        ridareamax=find([stacks(k,1).Framestats(ln,1).stats.Area]>250);        ridHint=find([stacks(k,1).Framestats(ln,1).Hstats.MeanIntensity]<meanmeancat);         ridall=cat(2,ridareamax,ridHint,ridareamin);        ridall=unique(ridall);        stacks(k,1).Framestats(ln,1).stats(ridall)=[];        stacks(k,1).Framestats(ln,1).Hstats(ridall)=[];         catround=cat(1,stacks(k,1).Framestats(ln,1).stats.Roundoid);        ridix=find(catround(:,1)<15 | catround(:,1)>(xpix-15));        ridy=find(catround(:,2)<15 | catround(:,2)>(ypix-15));        ridagain=unique(cat(1,ridix,ridy));        stacks(k,1).Framestats(ln,1).stats(ridagain)=[];        stacks(k,1).Framestats(ln,1).Hstats(ridagain)=[];          % defining a square whose center is centroid of nucleus from which area        % to extract mean cytoplsamic intensity          for m=1:length(stacks(k,1).Framestats(ln,1).stats)            xm=(stacks(k,1).Framestats(ln,1).stats(m).Roundoid(1,1)-13):(stacks(k,1).Framestats(ln,1).stats(m).Roundoid(1,1)+12);            %             kx=find(xm<1);xm(kx)=[];            %             kxx=find(xm>xpix);xm(kxx)=[];            ym=(stacks(k,1).Framestats(ln,1).stats(m).Roundoid(1,2)-13):(stacks(k,1).Framestats(ln,1).stats(m).Roundoid(1,2)+12);            %             ky=find(ym<1);ym(ky)=[];            %             kyy=find(ym>ypix);ym(kyy)=[];            ab=numel(xm);bb=numel(ym);            selec=zeros(ab*bb,2);            z=1;            if ab>=bb                for no=1:ab                    for p=1:bb                        Al=[xm(no) ym(p)];                        selec(z,:)=Al;                        z=z+1;                    end                end            else                for no=1:bb                    for p=1:ab                        Al=[xm(p) ym(no)];                        selec(z,:)=Al;                        z=z+1;                    end                end            end            stacks(k,1).Framestats(ln,1).stats(m).Selection=selec;            stacks(k,1).Framestats(ln,1).stats(m).Scyto=zeros(ab*bb,1);            for w=1:(ab*bb)                stacks(k,1).Framestats(ln,1).stats(m).Scyto(w)=GScytotemp(stacks(k,1).Framestats(ln,1).stats(m).Selection(w,2),stacks(k,1).Framestats(ln,1).stats(m).Selection(w,1));            end            stacks(k,1).Framestats(ln,1).stats(m).Scytor=reshape(stacks(k,1).Framestats(ln,1).stats(m).Scyto,ab,bb);            stacks(k,1).Framestats(ln,1).stats(m).Scyton=stacks(k,1).Framestats(ln,1).stats(m).Scyto;            nul=find([stacks(k,1).Framestats(ln,1).stats(m).Scyton] ==0);            stacks(k,1).Framestats(ln,1).stats(m).Scyton(nul)=[];            stacks(k,1).Framestats(ln,1).stats(m).Scytomean=mean(stacks(k,1).Framestats(ln,1).stats(m).Scyton);            stacks(k,1).Framestats(ln,1).stats(m).NCRatio=stacks(k,1).Framestats(ln,1).stats(m).MeanIntensity/stacks(k,1).Framestats(ln,1).stats(m).Scytomean;        end     endendtoc---------------------------------------------------------------------------------------2- Cleaning data% keep only nuclei with the biggest area from the different slices (meaning% that it's in the focus plan of this given slice) and rearrange the stats% to a new structure in=input('how many slices?');w=1;ambig=zeros(100,5);for i=1:length(stacks)    for a=1:(in-1)                for j=a+1:in            remove=cell({});            z=1;            for k=1:length(stacks(i,1).Framestats(a,1).stats)                roundcat=cat(1,stacks(i,1).Framestats(j,1).stats.Roundoid);                indx=ismember(roundcat(:,1),stacks(i,1).Framestats(a,1).stats(k,1).PixelList(:,1));                indy=ismember(roundcat(:,2),stacks(i,1).Framestats(a,1).stats(k,1).PixelList(:,2));                same=indx.*indy;                indf=find(same);                if numel(indf)>1                    zeros(w,1:5)=[i a k i j];                    w=w+1;                end                if numel(indf)==1                    if stacks(i,1).Framestats(a,1).stats(k,1).Area>=stacks(i,1).Framestats(j,1).stats(indf,1).Area                        remove{z,1}=[indf j];                        z=z+1;                    else                        remove{z,1}=[k a];                        z=z+1;                    end                end            end                        remove=cell2mat(remove);            for p=1:in                if isempty(remove)==0                    [clr clc]=find(remove(:,2)==p);                    stacks(i,1).Framestats(p,1).stats(remove(clr,1))=[];                    stacks(i,1).Framestats(p,1).Hstats(remove(clr,1))=[];                end            end        end    end    %     for m=1:in    %         ridareamax=find([stacks(i,1).Framestats(m,1).stats.Area]>300);    %         stacks(i,1).Framestats(m,1).stats(ridareamax)=[];    %         stacks(i,1).Framestats(m,1).Hstats(ridareamax)=[];    %     endendfor q=1:length(stacks)    cleanstacks=struct([]);    cleanstacks=cat(1,stacks(q,1).Framestats(:,1).stats);    fcleanstacks=['cleanstacks' int2str(q-1) '=cleanstacks'];    eval(fcleanstacks);end %function removecell(src,evnt,sc)%global z%                            if evnt.Character=='r'%                                remove{z,1}=[k a];%                                z=z+1;%                            elseif evnt.Character=='b'%                                remove{z,1}=[indf(sc) j];%                                    z=z+1;%end   ------------------------------------------------------------------------3- Tracking cells%this script/function should be able to track cells from stats from the%getstats function% Double check whether stats.Roundoid (round of stats.Centroid) effectively% exist. If not, should be replaced by stats.Centroid  %% concatenate Roundoid coordinates and NCratio for each frame      tot=input('how many frames total?');ask=input('how many starting frames?');mf=input('minimum track?');zslice=input('zslice interval (microns)?');%frate=input('frame rate (minutes)?');fact=zslice/2*1.42;mdist=5;ticncells=zeros(tot,1);for k=0:tot    kn=k+1;        stemp=['statstemp=cleanstacks' int2str(k)];    eval(stemp);    ncells(kn)=length(statstemp);    numb=zeros(length(statstemp),1);    numb(:,1)=k;    catR=cat(1, statstemp.Centroid); catNC=cat(1,statstemp.NCRatio);catslice=cat(1,statstemp.Slice);    cattemp=cat(2,numb,catR,catslice,catNC);    vcat=['cat' int2str(k)];    assignin('base',vcat,cattemp);end%% preallocate the array with cells in the first frame (stats0)cellsize=max(ncells);celltrack(cellsize,ask)=struct('cell',[]);for dd=1:cellsize    for ee=1:ask;    celltrack(dd,ee).cell(1:ask,1:5)=0;    ko=find(celltrack(dd,ee).cell==0);    celltrack(dd,ee).cell(ko)=NaN;    endend for lo=1:ask    fstatstart=['statstart=cleanstacks' int2str(lo-1)];    eval(fstatstart);    fcat=['cattemp=cat' int2str(lo-1)];    eval(fcat);    for pre=1:length(statstart)        celltrack(pre,lo).cell(lo,1:5)=cattemp(pre,:);        celltrack(pre,lo).cell(lo,6)=0;    end    %compute the distance of each cell from a frame to all the cells from the    %next frame    for frame=1:tot        if frame<lo            continue        end        fcat=['scat=cat' int2str(frame)];        eval(fcat);        for cc=1:size(celltrack,1)            if isempty(celltrack(cc,lo).cell)                break            end            if isnan(celltrack(cc,lo).cell)                break            end        end        for c=1:(cc-1)            mattemp=zeros(length(scat),1);            z=1;            for i=1:length(scat)                d=sqrt((celltrack(c,lo).cell((frame),2)-scat(i,2))^2+(celltrack(c,lo).cell((frame),3)-scat(i,3))^2+((celltrack(c,lo).cell((frame),4)-scat(i,4))*fact)^2);                mattemp(i)=d;                z=z+1;            end                    %get the minimal distance, if <10 pixels, add the cell of the next            %frame to the celltrack            [val,ind]=min(mattemp);            if val<mdist            celltrack(c,lo).cell((frame+1),1:5)=scat(ind,1:5);            celltrack(c,lo).cell((frame+1),6)=val;            elseif val>=mdist                celltrack(c,lo).cell((frame+1),:)=0;            end        end    endend%% clean the data by removing all cells that persist less than input number (mf) of framesfor lo=1:ask    for i=1:size(celltrack,1)        [row,col]=find(celltrack(i,lo).cell(:,2)==0);        celltrack(i,lo).cell(row,:)=[];        [rown coln]=find(isnan(celltrack(i,lo).cell));        celltrack(i,lo).cell(rown,:)=[];    end    for k=1:size(celltrack,1)        m=size(celltrack(k,lo).cell,1);        if m<mf        celltrack(k,lo).cell=[];        end    endendtoc -------------------------------------------------------------------------4- Remove duplicated cells%this code to remove tracked cells identified in previous frames%and creates trackfinal structure array! % starts from celltrack structure array from trackallatonce %% remove cells present in the previous frame (starting from the last% frame)function trackfinal=removedupcells(celltrack) totframe=size(celltrack,2);totcells=size(celltrack,1);for ii=totframe:-1:2    for all=1:totcells        for allp=1:totcells            if isempty(celltrack(all,ii).cell) || isempty(celltrack(allp,(ii-1)).cell)            continue            end        ff=ismember(celltrack(all,ii).cell(1,2:3),celltrack(allp,(ii-1)).cell(2,2:3));        if sum(ff)==2            celltrack(all,ii).cell=[];        end        end    endend%% and create a new structure - trackfinal- where only non empty cells are put infor count=1:size(celltrack,2)    fcount=genvarname(['count' int2str(count)]);    rcount=0;    for l=1:size(celltrack,1)        if isempty(celltrack(l,count).cell)            continue        end        rcount=rcount+1;    end    eval([fcount '=rcount'])endtrackfinal(count1,size(celltrack,2))=struct('cell',1);for ll=1:size(celltrack,2)    row=1;    for kk=1:size(celltrack,1)        if isempty(celltrack(kk,ll).cell)           continue        end        trackfinal(row,ll).cell=celltrack(kk,ll).cell;        row=row+1;    endendtrackfinal(end,end).cell=[];  -------------------------------------------------------------------------------------5- Manually define margin position using a GUI function YSLposv2(prostacks) global pos count snb in postempwarning offin=input('How many points?');  nb=input('how many frames?');postemp=[]; for j=1:nb    disp(j)    snb=int2str(j);%     if j<10%         tag=['0' snb];%     elseif j>=10%         tag=snb;%     end    pos=zeros(in,2);    count=0;    I=prostacks(:,:,j);    figure('deletefcn',{@closing,pos, snb},'pointer', 'crosshair');    imshow(I);    ax=gca;    axn=axes('buttondownfcn',{@click,count, pos,in},'position',get(ax,'position'),'XLim',get(ax,'XLim'),'YLim',get(ax,'YLim'),'Ydir','reverse','color','none');    hold(gca,'on');    if isempty(postemp)==0        scatter(gca,postemp(:,1),postemp(:,2),'or', 'hittest','off');    end    pause;endend      function click(gcbo,eventdata,count, pos, in)    global count pos    count=count+1;    z=get(gca,'currentpoint');    pos(count,:)=z(1,1:2);    if count==in        close;    end    end     function closing(gcbo,eventdata,pos, snb)        global pos snb postemp        postemp=pos;        fname=['Margin' snb];        assignin('base',fname,pos);    end  ---------------------------------------------------------------------------------6- Measure distance of each cell at each time point from the margin        % create structure array for margin variable, get fit and measure distance% from margin nb=input('how many frames?');YSLstats=struct([]);for i=1:nb    mtemp=['Margintemp=Margin' int2str(i)];    eval(mtemp);    YSLstats(i,1).coord=Margintemp;    YSLstats(i,1).fit=fit(Margintemp(:,1),Margintemp(:,2),'poly2');end%% compute distance from margin %for k=1:size(trackfinal,2)for k=1:nb    for l=1:size(trackfinal,1)        if size(trackfinal(l,k).cell,1)>nb-k            n=nb-k;        elseif size(trackfinal(l,k).cell,1)<=nb-k            n=size(trackfinal(l,k).cell,1);        end                for h=1:n            trackfinal(l,k).cell(h,7)=(YSLstats((h+k-1),1).fit(trackfinal(l,k).cell(h,2))-trackfinal(l,k).cell(h,3))*425.1/512;        end           endend  
